# Mesenchymal Stromal Cells‐Mediated Tumor‐Targeted Delivery of Oncolytic Bacteria Combined With Immune Checkpoint Blockade Against Melanoma

**DOI:** 10.1002/mco2.71142

**Published:** 2026-11-13

**Authors:** Yanning Wang, Tianyun Gao, Feifei Huang, Yaping Wang, Mingmei Yang, Congwang Xu, Dong Chen, Shuo Liu, Yuanyuan Xie, Yong Liu, Bin Wang

**Affiliations:** ^1^ Clinical Stem Cell Center Nanjing Drum Tower Hospital The Affiliated Hospital of Nanjing University Medical School Nanjing China; ^2^ Clinical Stem Cell Center Nanjing Drum Tower Hospital Nanjing University of Chinese Medicine Nanjing China; ^3^ Department of Laboratory Medicine Nanjing Drum Tower Hospital Clinical Medical College of Traditional Chinese and Western Medicine Nanjing University of Chinese Medicine Nanjing China

**Keywords:** engineered MSCs, PD1 nanobody, VNP20009, tumor‐targeted delivery, immune activation

## Abstract

Oncolytic bacterial therapy has huge application potential for cancer treatment due to its tumor‐targeting capabilities and immune microenvironment activation. However, the severe adverse effects of oncolytic bacteria limit their clinical applications. Enhancing biosafety while maintaining the efficacy of oncolytic bacteria is essential for advancing the clinical translation of live microbial cancer therapies. Here, we construct a mesenchymal stromal cell (MSC)‐mediated tumor‐targeted delivery system for *Salmonella typhimurium* VNP20009 (MSC‐VNP), which significantly enhances the intertumoral enrichment and safety of VNP. This benefit arises from the tumor‐homing chemotaxis of MSCs and the delayed release of intracellular VNP. To improve the therapeutic effect, we construct engineered MSCs that sustainably secrete PD‐1 nanobodies (MSC‐PD1nb) to treat melanoma in combination with MSC‐VNP. This combination therapy improves antitumor efficacy in a mouse melanoma model by increasing antitumor effector cells (CD8 ^+^ T effector cells) and reducing protumor effector cells (CD4 ^+^ T regulatory cells). These data indicate that MSC‐mediated combination immunotherapy offers a promising antitumor immune strategy, providing a prospect for expanding the therapeutic application of oncolytic bacteria in cancer.

## Graphical abstract

Most existing oncolytic bacterial therapies are limited by systemic toxicity and poor tumor accumulation. We engineered an MSCs‐mediated combination therapy utilizing MSCs‐VNP for tumor‐targeted bacterial delivery and MSC‐PD1nb for sustained local secretion of PD1 nanobodies. (1) MSCs‐VNP release VNP within tumors to induce apoptosis while minimizing off‐target toxicity, whereas MSC‐PD1 b blocks the PD1‐PDL1 axis. (2) This synergistic strategy remodels the immunosuppressive microenvironment by promoting M1 macrophage polarization, DC maturation, and CD8^+^ T cell activation while reducing M2 macrophages and Tregs, offering a promising tumor‐specific immunotherapy for melanoma.

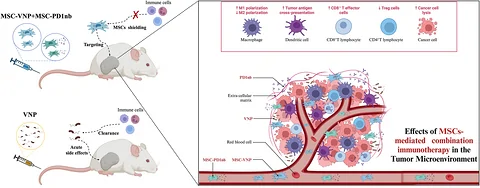

## 1 Introduction

The tumor microenvironment (TME) imposes critical barriers to conventional immunotherapies, including impaired T cell infiltration and pronounced immunosuppressive signaling [1, 2, 3, 4]. Consequently, given the immunosuppression and complexity of the TME, treatments targeting a single effector, such as immune checkpoint blockers (ICBs), like PD1/PDL1, are unlikely to completely eliminate tumors [5, 6]. To overcome these limitations, oncolytic bacteria have emerged as a promising strategy due to their ability to preferentially colonize hypoxic tumors and activate antitumor immunity [7, 8, 9, 10, 11]. Among these, attenuated *Salmonella typhimurium* VNP20009 (VNP) exhibits potent antitumor activity, yet its clinical application is limited by substantial toxicity and off‐target effects, as evidenced by the termination of a phase I trial [12, 13].

Numerous studies have explored methods to enhance the biosafety of bacterial therapies, such as coating polydopamine [14], alginate [15], and cell membranes [16, 17] on strain surfaces, to camouflage bacteria. These strategies enhanced strain biocompatibility by transiently reducing immunogenicity. However, bacterial proliferation leading to the shedding of modification layers still poses a potential safety concern. Thus, enhancing biocompatibility while preserving tumor immunity activation and colonization ability remains a significant challenge in oncolytic bacteria‐based antitumor therapy.

Recently, cell carrier systems have emerged as a promising field of research. These systems contain various cell types [18, 19, 20], such as immune cells (e.g., neutrophils, macrophages, natural killer cells, and T lymphocytes) and erythrocytes, which offer significant advantages over polymer‐based materials, including enhanced capacity to cross physiological barriers, extended circulation times, and superior biocompatibility, because these cells are “self” materials. Recent studies have highlighted the cell‐mediated drug delivery systems as a promising approach to enhance the biosafety of oncolytic bacteria in vivo. This innovative strategy utilizes cell carriers (e.g., neutrophils and macrophages) for the targeted delivery of VNP [21, 22, 23]. This approach achieves a balance between the safety and efficacy of VNP, using neutrophils and macrophages as carriers to shield VNP and release it within the tumor core. However, the limited availability, immune rejection, and inflammatory cytokine storm limit the clinical application potential of bacterial delivery strategies based on such cells [24].

Due to their low immunogenicity, abundant availability, and good chemotaxis, mesenchymal stromal cells (MSCs) have attracted substantial interest as natural delivery vehicles for therapeutic molecules and proteins, including interleukin‐10 (IL‐10), basic fibroblast growth factor (bFGF), and monoclonal antibodies (mAbs) [25, 26, 27]. Additionally, MSCs’ strong tumor‐homing capabilities make them suitable for cancer drug delivery, demonstrating significant antitumor effects in preclinical studies and potential for clinical application [28, 29, 30, 31]. Therefore, a hypothesis is that MSCs‐mediated tumor‐targeted delivery of VNP (MSCs‐VNP) could leverage MSCs’ tumor‐homing properties and control the release of intracellular strains after infiltrating into tumors, minimizing adverse reactions from direct VNP administration. Furthermore, our previous studies demonstrated that transplanted engineered MSCs expressing mAbs (MSCs‐mAbs) could continuously secrete mAbs into serum in vivo, achieving more stable antibody levels than the standalone mAbs administration group [27]. Accordingly, we assume that the PD1 antibody‐engineered MSCs would infiltrate into the tumor and secrete the PD1 nanobodies (PD1nb) constantly, thereby enhancing PD1nb's antitumor therapeutic effect. Meanwhile, since subcutaneous tumors are more difficult for cell carriers to reach than orthotopic tumors, we chose a melanoma model by subcutaneous inoculation to investigate the efficacy of the MSC‐mediated VNP delivery system in this study.

This study presents, for the first time, an MSC‐mediated VNP delivery system for tumor therapy. This novel strategy utilizes the tumor‐homing properties of MSCs and the tumor‐killing effects of VNP, ultimately balancing VNP safety and antitumor efficacy. To further boost the therapeutic efficacy of this VNP delivery system, we combined engineered MSCs secreting PD1nb (MSC‐PD1nb), significantly enhancing therapeutic outcomes in melanoma models.

## 2 Results

### 2.1 Human MSCs Can Function as Cell Carriers of VNP

Initially, we co‐cultured the bacteria with MSCs to generate VNP‐encapsulated MSCs (MSCs‐VNP). Extracellular VNPs were eliminated with 50 µg/mL gentamicin for 60 min (Figure S1A). Additionally, the plate counting method was employed to determine the number of viable VNPs following MSC encapsulation, which identified optimal co‐culture conditions. The results indicated that a higher encapsulation efficiency was achieved under a multiplicity of infection (MOI) of 100 and serum‐free co‐culture conditions (Figure S1B–E). As shown by fluorescence microscopy (Figure 1A), MSCs successfully encapsulated VNP during co‐culture. Moreover, an image from a transmission electron microscope displayed an intuitive visualization of intracellular VNP (Figure 1B). Flow cytometry was also employed to detect VNP strains inside MSCs, further confirming the intracellular loading of VNP by the MSCs (Figure 1C,D). Based on these findings, we successfully generated MSCs‐VNP.

**FIGURE 1:**
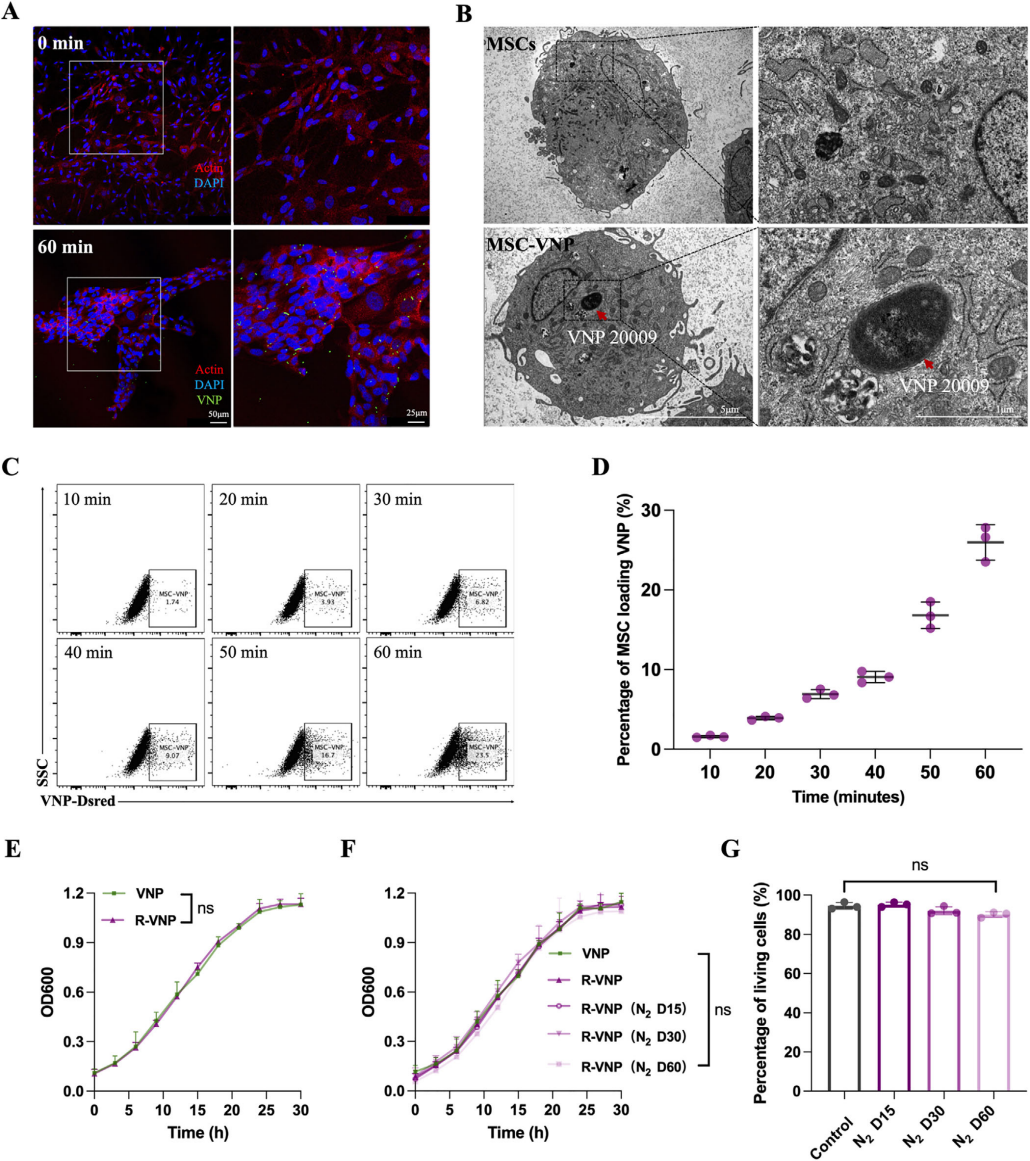
Preparation of VNP‐encapsulated MSCs. (A) Fluorescent images of MSCs‐VNP (co‐cultured for 0min or 60 min, MOI 100); nuclei (DAPI, blue), actin (red), VNP (FM1‐43, green). Scale bar = 50 µm (left) or 25 µm (right). (B) Representative transmission electron micrographs of MSCs‐VNP (co‐cultured for 60 min, MOI 100); The arrow in the image points to the intracellular VNP. Scale bar = 5 µm (left) or 1 µm (right). (C, D) The loading efficiency of VNP (FM1‐43, green) by MSCs at different time points measured by FACS (*n* = 3). (E) Growth curves of normal VNP compared with the MSCs‐released VNP strain (R‐VNP) in liquid LB medium (*n* = 3). (F) Growth curves of the MSCs‐released VNP strain (R‐VNP) after cryopreservation in liquid nitrogen for 15/30/60 days (*n* = 3). (G) Comparison of the viability of MSCs after loading VNP and cryopreservation in liquid nitrogen for 15/30/60 days; the media were supplemented with gentamicin (*n* = 3).

To investigate the mechanism underlying MSC encapsulation of VNP, we co‐cultured MSCs and macrophages with inactivated VNP, respectively (Figure S2A–C). Fluorescence imaging showed that the cells could not encapsulate inactivated VNP. Therefore, the successful VNP encapsulation of MSCs primarily depended on bacterial infection of the cells rather than active phagocytosis by the cells. The encapsulating efficiency improved with extended incubation time (Figure 1C,D; Figure S1D–F). Over time, the number of VNP strains loaded in MSCs increased (Figure S1D,E), although the survival rate of MSCs gradually decreased (Figure S3A), likely due to persistent VNP stimulation. In summary, a co‐culture time of 1 h was chosen for subsequent experiments due to its satisfactory loading efficiency (≈114 strains per 100 cells) and high cell survival rate (>90%). Similarly, the viability of MSCs after loading VNP for 24 h was also assessed (Figure S3B). The results indicated that at least 50% of the MSCs‐VNP (co‐culture 1 h) viability was maintained after 24 h, by which time most intravenously administered MSCs had accumulated at the tumor site [32], ensuring the effectiveness of MSCs tumor‐targeted delivery. The intracellular strain maintained activity comparable to that of untreated free VNP strain (Figure 1E). Additionally, MSCs‐VNP demonstrated robust bacterial proliferation and cell viability after liquid nitrogen cryopreservation (Figure 1F,G). Importantly, the antitumor efficacy of VNP remained unimpaired (Figure S4). These findings lay the foundation for effective antitumor MSC‐mediated tumor‐targeted delivery of bacteria.

### 2.2 Characterization of MSCs‐VNP

To evaluate the chemotactic properties of MSCs after encapsulating VNP, Transwell chemotaxis assays were conducted. Tumor cells and MSCs‐VNP or untreated MSCs were seeded in the lower and upper chambers of an 8 µm aperture Transwell chamber, respectively (Figure 2A). After 12 h of incubation, the upper chambers were collected to count the cells (Figure 2B). As shown in Figure S5, the chemotactic capability of MSCs toward melanoma tumor cells (B16F10) remained unaffected. Similarly, the same trend was observed when co‐cultured with other tumor cell types, including HGC‐27 (human gastric cancer cells) and A549 (human lung cancer cells). To further elucidate the VNP release dynamics from MSCs‐VNP, we constructed engineered VNP strains expressing red fluorescent protein (DsRed), designated as VNP‐DsRed (Figure S6). MSCs and VNP‐DsRed were co‐cultured in medium, and the release dynamics of VNP‐DsRed from MSCs‐VNP were continuously monitored using a live‐cell imaging workstation over a 24 h period. To prevent interference from the self‐proliferation of released VNP‐DsRed, a low concentration of gentamicin was added to the medium to inhibit VNP growth without killing the bacteria. Notably, delayed VNP release was observed at 9 h, with complete release occurring by 18 h (Figure 2C; Figure S7 and Video S1). The release of VNP may be attributed to the sustained motility and proliferation of intracellular strains. Meanwhile, the supernatant was collected, and the number of bacterial colonies released from the cells was counted using plate counting (Figure 2D,E). Consistent with previous results, MSCs‐VNP also exhibited delayed strain release, with the number of released VNP increasing over time. Additionally, to assess whether VNP could maintain its antitumor ability after being released from MSCs, in vitro co‐culture experiments were conducted with tumor cells (Figure 2F,G). The released VNP strains effectively induced apoptosis in tumor cells. Meanwhile, VNP released from MSCs‐VNP exhibited comparable growth profiles and antitumor efficacy to wild‐type VNP under a simulated tumor microenvironment (low pH and hypoxia) (Figure S8A,B). Therefore, it is reasonable to assume that these released VNP strains have the potential to promote tumor regression by directly killing tumor cells and remodeling the tumor microenvironment. In summary, we confirmed that MSCs can act as natural biological carriers of bacteria, enabling targeted delivery to tumor cells and delayed release of VNP strains to execute classic antitumor efficacy.

**FIGURE 2:**
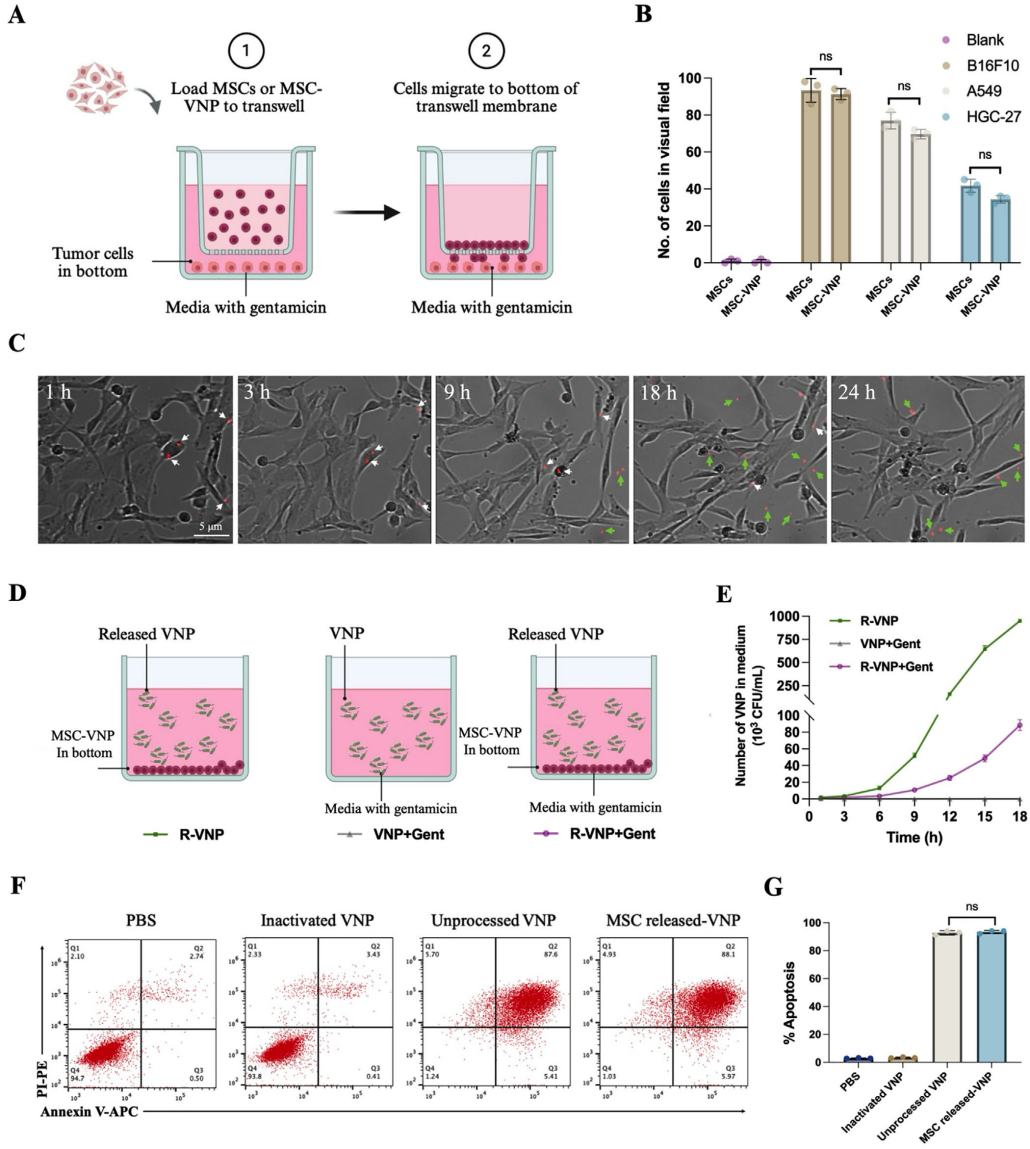
In vitro tumor chemotaxis of MSCs‐VNP and VNP release behavior or tumoricidal activity. (A) The experimental diagram of the chemotactic assay: the lower chambers were seeded with tumor cells; MSCs or MSCs‐VNP were added to the upper chambers (8 µm); 12 h of incubation with 50 µg/mL gentamicin. (B) The chemotactic number of cells (*n* = 3). (C) Representative brightfield images of the delayed release of the intracellular VNP‐DsRed strain from MSCs are shown; white arrows point to intracellular VNP‐DsRed, and green arrows point to extracellular VNP‐DsRed; medium containing 10 µg/mL gentamicin. Scale bar = 5 µm. (D) The experimental diagram of the release assays: the MSCs‐VNP were incubated in medium with or without 10 µg/mL gentamicin, and the gentamicin‐treated free VNP as the control group. (E) The release profiles of VNP strains from MSCs‐VNP over time (*n* = 3). (F, G) The apoptosis of B16F10 after incubation with inactivated VNP, unprocessed VNP, MSC‐released VNP, or none for 16 h (*n* = 3).

### 2.3 MSCs‐VNP Promotes VNP Strains Infiltration and Proliferation in Tumors

MSCs naturally exhibit tumor‐targeted chemotaxis tendencies [33, 34]; thus, we investigate whether MSCs‐VNP could successfully deliver VNP into a subcutaneous melanoma inoculation. First, to clearly trace the transplanted MSCs‐VNP in vivo, MSCs expressing enhanced green fluorescent protein (MSCs‐EGFP) were loaded with VNP‐DsRed and intravenously injected into melanoma tumor‐bearing mice. MSCs‐EGFP (green) and VNP‐DsRed (red) infiltration were then observed in resected tumors at various time points (Figure 3A). The flow cytometry results indicated that MSCs‐EGFP infiltrated into the solid tumor as early as 18 h after transplantation (Figure 3B,C), while slice scanning revealed VNP‐FM1‐43 tumor colonization at the same time point (Figure S9A), coinciding with MSC colonization. The number of tumor‐infiltrating VNP‐FM1‐43 was significantly increased at 48 h, indicating that released VNP from MSCs‐VNP had rapid proliferation in the tumor. Notably, compared with free VNP, VNP released from MSCs‐VNP exhibited preferential enrichment within the tumor core, although VNP from both groups were ultimately cleared by immune cells by 2 weeks postadministration (Figure S9B,C). To further visualize the dynamic changes of MSCs‐VNP accumulation within tumors, an in vivo situ cell dynamic imaging system was used to analyze the biodistribution of MSCs‐EGFP‐encapsulated VNP‐DsRed at different time points (Figure 3D; Videos S2–S4). In the MSCs‐VNP group, VNP‐DsRed was observed in melanoma tissue at 18 h, whereas in the VNP group, VNP‐DsRed remained in blood vessels. At 24 and 48 h, VNP colonization and proliferation were observed in the tumor tissue in both groups. Therefore, owing to the natural tumor infiltration capability of MSCs at 18 h, MSCs‐VNP facilitate earlier VNP infiltration into tumors. Consistently, this finding was further validated by flow cytometry analysis (Figure 3E,F).

**FIGURE 3:**
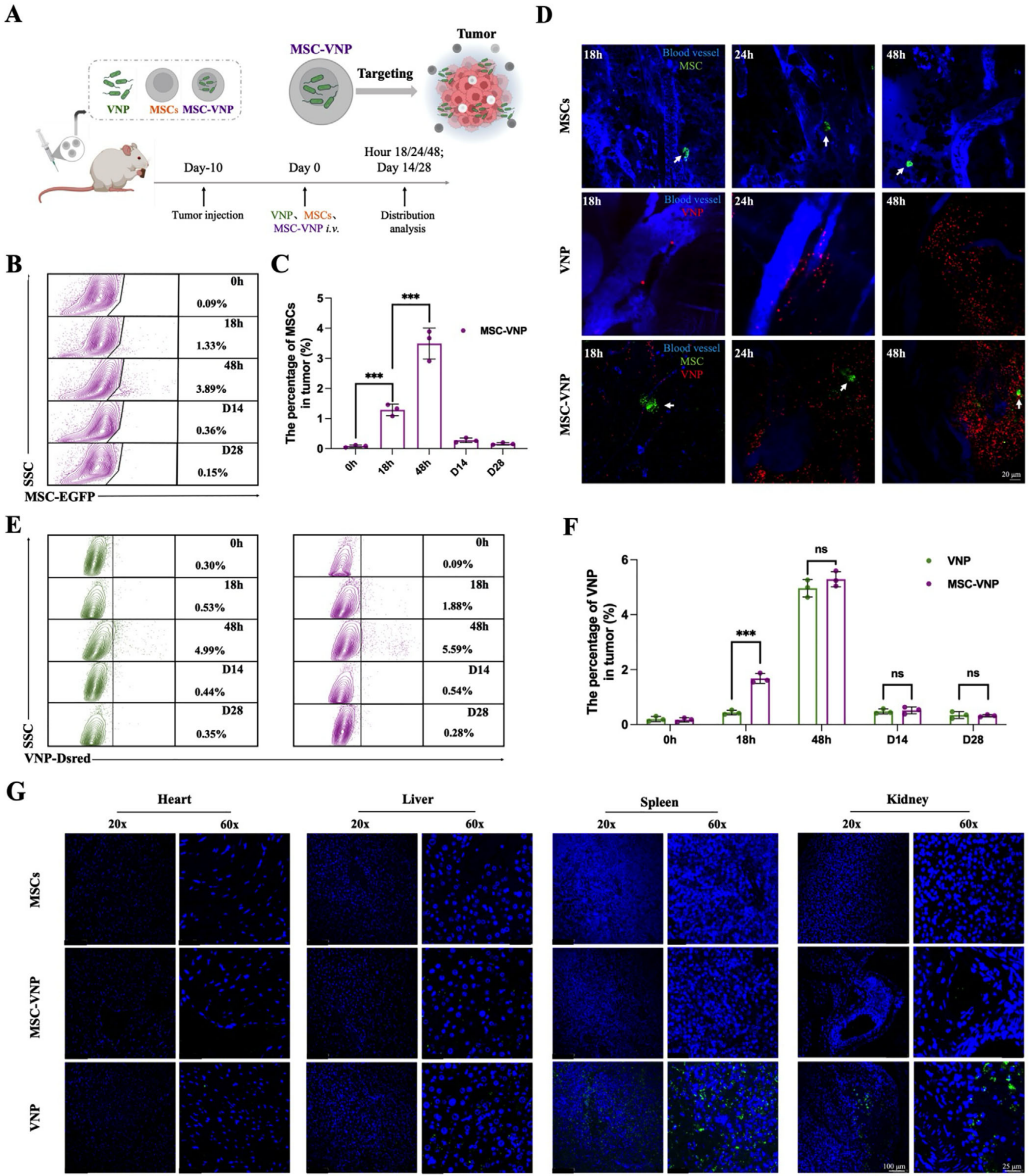
MSCs‐VNP promotes intratumor enrichment of VNP in vivo. (A) Schematic of tracing the transplanted MSCs‐VNP in a mouse melanoma model (MSCs‐EGFP: 5 × 10^5^ cells per mouse, VNP‐DsRed strains: 5.7 × 10^5^ CFU per mouse, MSCs‐VNP: 5 × 10^5^ cells per mouse). (B, C) Representative FACS plots and quantified data of infiltrated MSCs‐EGFP in tumors at different time points (*n* = 3). (D) Representative images of melanoma models after different treatments at different time points by an in vivo situ cell dynamic imaging system (tumor blood vessels: blue, VNP‐DsRed: red, MSC‐EGFP: green). The arrow in the image points to the MSC‐EGFP. Scale bar = 20 µm. (E, F) Representative FACS plots and quantified data of infiltrated VNP‐DsRed in tumors at different time points (*n* = 3). (G) The organ burden of VNP at 48 h after administration in the melanoma model (nuclei: DAPI staining [blue], VNP: FM1‐43 [green]). Scale bar = 100 µm (left) or 25 µm (right).

The above results reveal that MSCs‐VNP are preferentially enriched in tumors, whereas free VNP remain relatively confined to blood vessels. Based on these observations, we speculate that the MSCs‐VNP delivery system has the potential to reduce VNP strain titers in normal organs. To verify this hypothesis, immunofluorescence was first employed to observe VNP colonization across various organs (Figure 3G; Figure S10). Results showed fewer VNP were observed in the spleen, kidneys, and lungs after MSCs‐VNP transplantation, while direct VNP administration led to significant accumulation in all organs except the heart. Concurrently, flow cytometry further demonstrated that VNP titers in normal organs were low following MSCs encapsulating VNP (Figure S11A,B). Plate counting provided a more intuitive comparison of VNP infiltration levels, and results showed that there were far fewer VNP colonies in the spleen, lungs, heart, and liver in the MSCs‐VNP group than in the VNP group (Figure S11C,D). Notably, no significant differences in intertumoral bacterial titers within each group. This observation is reasonable, as MSCs‐VNP continuously release intracellular VNP and preferentially colonize tumors; strains usually proliferate rapidly in tumors [17, 35], leading to a rapid increase in intertumoral bacterial titers at later stages. Consequently, significant differences may not be easily observed with simple VNP administration. In summary, the key advantage of MSCs‐VNP is earlier tumor core enrichment and reduced off‐target toxicity.

### 2.4 MSCs‐VNP Markedly Reduce Biotoxicity Compared With Direct VNP Administration

Intravenous injection of bacteria leads to rapid and excessive immune activation due to surface antigens, resulting in side effects commonly associated with bacterial therapy such as weight loss and hepatosplenomegaly in tumor‐bearing mice [36]. To test this hypothesis that MSC camouflage might attenuate bacteria‐induced side effects, MSCs‐VNP or alone VNP were administered to healthy mice via tail vein injection, and the acute side effects commonly associated with bacterial therapy were assayed at day 3 postinjection (Figure 4A). The results indicated that following MSCs‐VNP administration, the rapid weight loss and splenomegaly induced by alone VNP injection in mice were significantly abolished (Figure 4B–D; Figure S12A). Liver injury markers, including aspartate aminotransferase (AST) and alanine aminotransferase (ALT) levels, were markedly increased after alone VNP injection, while the increased AST and ALT were significantly attenuated in the MSCs‐VNP group (Figure 4E). The hematoxylin and eosin (H&E) staining showed that alone VNP injection induced obvious pathological injury in spleen and liver tissues, but these lesions were reduced in the MSCs‐VNP group, with almost no significant abnormalities in other organs compared with the saline group (Figure S12B). Bacteria activate immune cells to produce a large amount of inflammatory factors [37], and there exists a correlation between the degree of inflammation observed in vivo and the number of Treg cells present in the blood [38]. Therefore, we further examined the levels of inflammatory response protein (C‐reactive protein, CRP) and Treg cell population in peripheral blood at day 3 postadministration (Figure 4F–H). The upregulation of CRP and Treg cells triggered by single VNP strain administration was no longer observed in the MSCs‐VNP group. Moreover, three mice died following VNP injection, whereas all mice treated with MSCs‐VNP survived until the end of the study period (Figure 4I). These results indicate that compared with single VNP strain administration, MSCs‐based camouflage protection effectively decreased the toxic side effects caused by bacterial therapy, underscoring the potential of MSCs‐mediated antitumor microbial delivery as a superior cancer‐targeted therapy. The long‐term side effects were assessed at days 14 and 28 postinjection. The results indicated that weight loss and splenomegaly induced by VNP alone were gradually alleviated (Figure S13A–C). Moreover, serum biochemistry and H&E staining revealed that no long‐term toxicity or immunotoxicity was detected in mice across various treatment groups by day 28 (Figure S13D,E, S14A–C, and S15). This may be due to the gradual clearance of the VNP over time (Figure 4J,K; Figure S16A–C). This notion was further corroborated by biodistribution analysis of VNP across various organs. By day 28, VNP was eliminated from all organs (Figure S16A–C).

**FIGURE 4:**
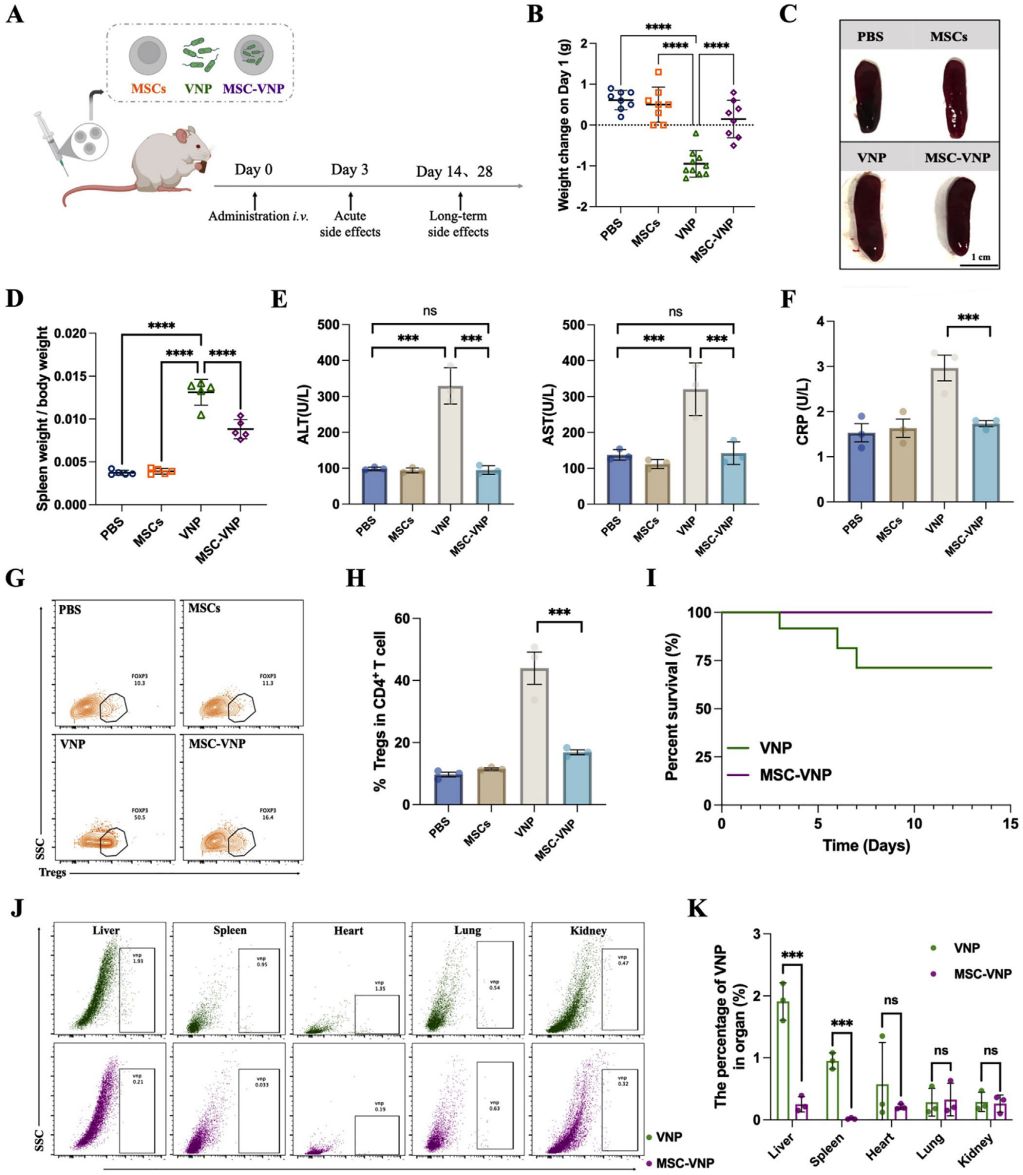
MSCs‐VNP significantly decreased the side effects of alone VNP administration. (A) Schematic of the safety assessment of mice after different treatments, including saline, VNP strains (5.7 × 10^5^ CFU per mouse), MSCs (5 × 10^5^ cells), and MSCs‐VNP (5 × 10^5^ cells per mouse). (B) Body weight changes 1 day after different treatments in healthy mice (*n* = 8 or 10). (C, D) Representative images of the spleen and ratio of spleen weight/body weight on day 3 after different treatments. Scale bar = 1 cm (*n* = 5). (E, F) Serum alanine transaminase (ALT), aspartate transaminase (AST), and C‐reactive protein (CRP) levels of mice on day 3 after different treatments (*n* = 3). (G, H) Representative FACS plots and the percentage of Tregs in the peripheral blood on day 3 after different treatments (*n* = 3). (I) Survival curves of the mice after different treatments (*n* = 8 or 10). (J, K) Representative FACS plots and quantified data 14 days after administration; organ burden of VNP‐DsRed in the healthy mouse (*n* = 3).

### 2.5 MSCs‐VNP Effectively Inhibit the Growth of B16F10 Tumor

To evaluate the anticancer efficacy of this MSCs‐mediated VNP delivery system, we administered MSCs‐VNP to melanoma‐bearing mice via intravenous injection (Figure 5A). The VNP dosage was determined based on the amount of VNP loaded by MSCs. Tumor growth curves showed that alone MSCs injection did not exhibit antitumor efficacy. In contrast, VNP administration alone significantly inhibited the progression of melanoma, reducing tumor volume by 36.2% compared with PBS and MSCs injection. The MSCs‐VNP administration also exhibited a significant blockage of tumor progression; however, no significant difference was observed between the VNP group and the MSCs‐VNP group (Figure 5B; Figure S17A,B). To more accurately assess the differences in tumor inhibition, tumors from each group were weighed. At day 7 after administration, the tumor weight of mice treated with MSCs‐VNP was only 35.4% of that in the saline‐treated group (Figure 5C), although no significant difference was observed compared with the VNP group. This lack of difference might be attributed to similar intertumoral VNP strain titers in both groups at later stages (Figure 3E,F). Notably, MSCs‐VNP treatment substantially extended survival times (Figure 5D). Furthermore, eosin (H&E) staining displayed more necrotic areas in tumors from mice treated with MSCs‐VNP or VNP administration alone (Figure 5E,F). Additionally, tumors in both the VNP and MSCs‐VNP groups exhibited pronounced apoptosis, with apoptotic areas corresponding to regions of VNP colonization in the tumor, suggesting that the antitumor effect of MSCs‐VNP is partly due to the lytic activity of VNP on tumor cells (Figure 5G; Figure S18). In summary, MSCs‐VNP not only improved the biosafety of VNP but also exhibited significant antitumor effects, enhancing its potential for clinical application.

**FIGURE 5:**
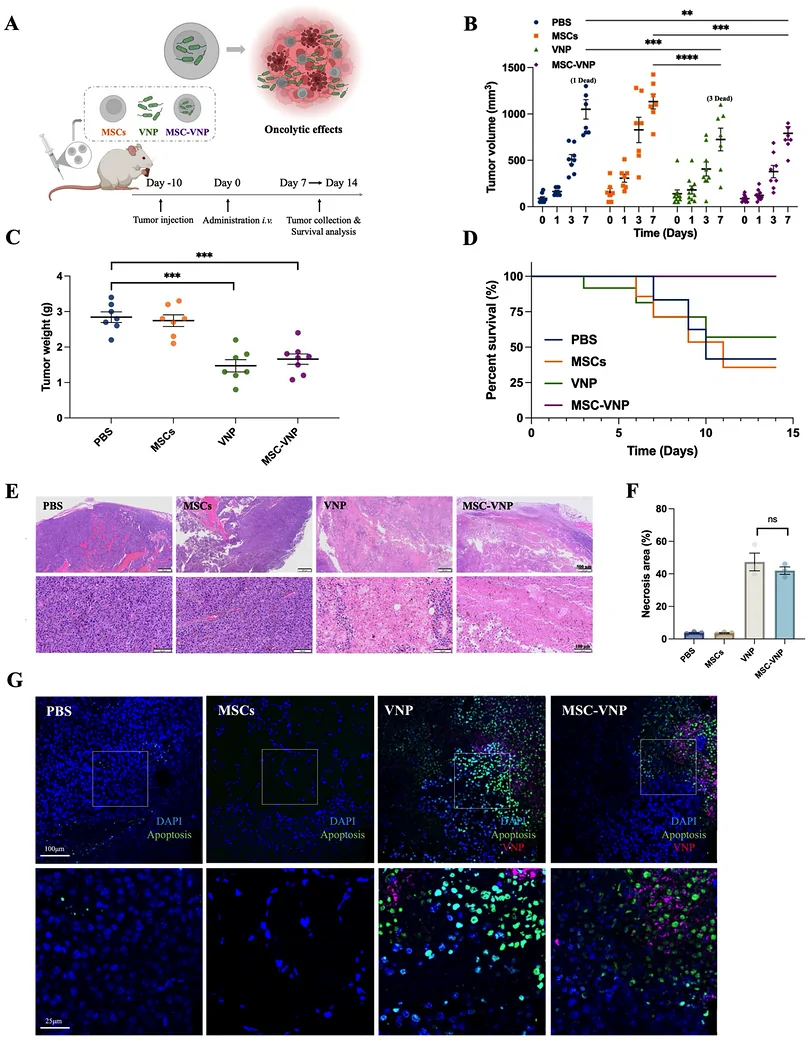
MSCs‐VNP effectively inhibit tumor growth. (A) Schematic diagram of the melanoma mouse model under different treatments, including saline, MSCs (5 × 10^5^ cells per mouse), VNP strains (5 × 10^5^ CFU per mouse), and MSCs‐VNP (5 × 10^5^ cells per mouse). (B) Tumor growth profiles of melanoma‐bearing mice after different treatments. (*n* = 7 or 8). (C) Tumor weight of melanoma‐bearing mice on day 7 after different treatments (*n* = 7 or 8). (D) Survival curves of melanoma‐bearing mice (*n* = 8). (E, F) Representative H&E images of the tumor and tumor necrosis proportion in different treatment groups at day 7. Scale bar = 500 µm (upper) or 100 µm (lower) (*n* = 3). (G) Representative fluorescence images of apoptosis staining in B16F10 tumor (green) after different treatments on day 7 (nuclei: DAPI [blue], VNP‐DsRed [red]). Scale bar = 100 µm (upper) or 25 µm (lower).

### 2.6 MSC‐PD1nb Synergistically Enhanced Antitumor Efficacy

To further enhance the therapeutic efficacy of our cell therapy strategy, and considering the two primary limitations of anti‐PD1 antibodies in clinical applications—namely, the requirement for continuous administration and off‐target effects—utilizing MSCs to steadily express anti‐PD1 antibodies may offer a synergistically improved antitumor efficacy. Consequently, we constructed an engineered MSC‐PD1nb cell line that can stably express and secrete PD1nb. As shown in Figure 6A, to ensure the intracellular expression and extracellular secretion of PD1nb, the expression plasmid (pcDNA3.1) was modified with the promoter EF‐1a, and the signal peptide sequence was fused to PD1nb. To verify the PD1nb function, 293T cells were transfected with the corresponding recombinant plasmids. The binding capacity of secreted PD1nb in the supernatant to PD1 protein was detected posttransfection. As expected, PD1nb secreted by 293T cells showed potent and specific binding to PD1 protein (Figure S19A). MSCs transfected with the EGFP plasmid were used to generate a vector control cell line; the recombinant plasmid was introduced into MSCs via electroporation, and the positive stable cell clones were screened out by verifying PD1nb secretion in the culture supernatant (Figure S19B). Herein, we obtained 20 MSC clones capable of successfully secreting PD1nb that recognizes PD‐1 protein. The PD1nb concentrations in the culture supernatant secreted by MSC clones ranged from 23.1 to 228.5 ng/mL. Western blotting verified that the engineered MSCs highly expressed and secreted PD1nb in vitro, with a nanobody size of 14 kDa (Figure 6B). Based on the concentration of PD1nb secreted by MSC clones, we selected the top‐performing MSC clone (clone NO. 18) for subsequent experiments. We then assessed the impact of MSC passage on PD1nb secretion (Figure S19C). As expected, the PD1nb secretion levels remained stable across MSC‐PD1nb passages.

**FIGURE 6:**
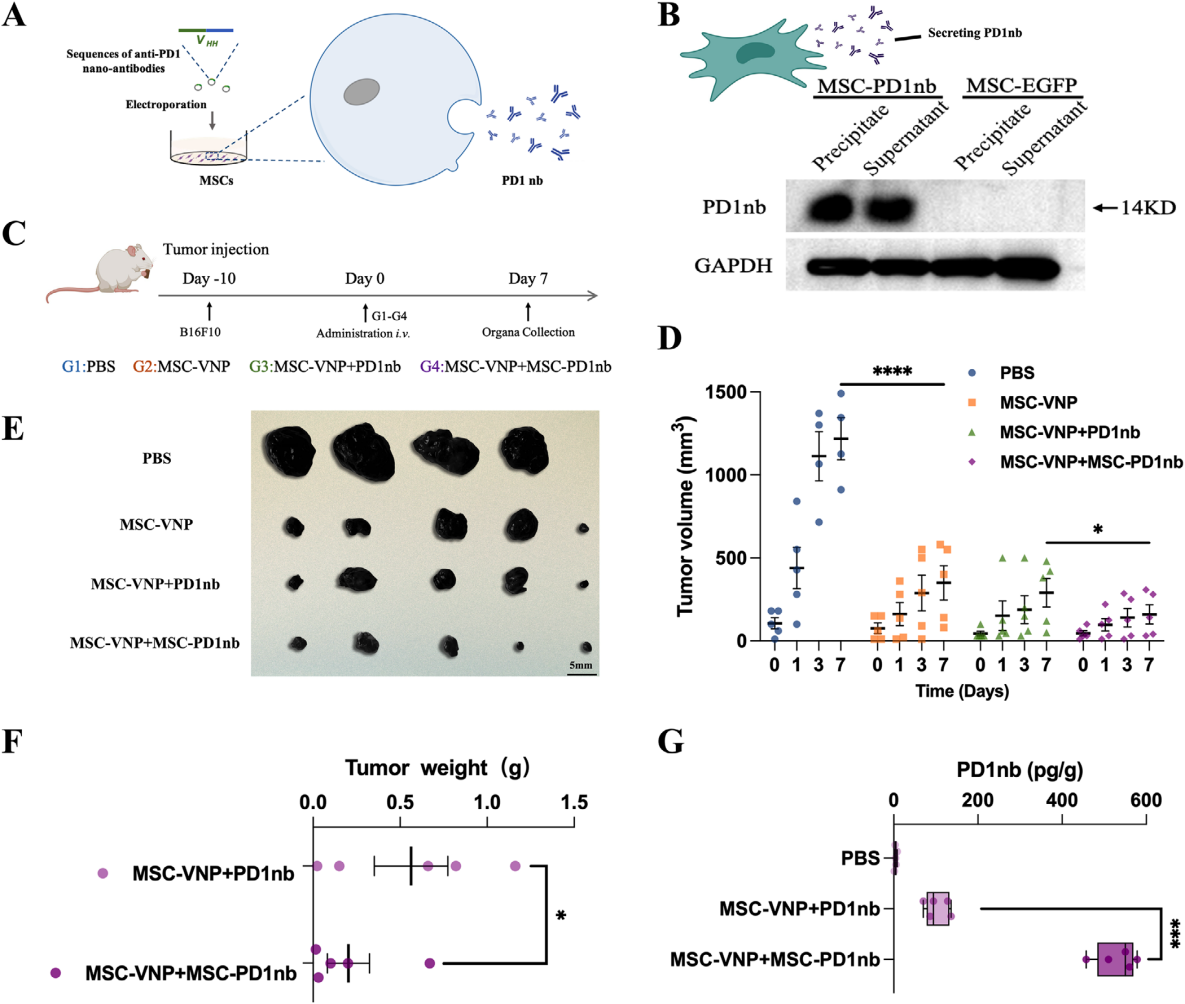
MSC‐PD1nb synergistically improves the antitumor effects of MSCs‐VNP. (A) Schematic of the preparation of MSC‐PD1nb. A plasmid containing the expression promoter EF1a, the protein signal peptide, and the target protein PD1nb was constructed. The plasmid was electrotransformed into the MSCs to obtain the engineered MSC‐PD1nb. (B) The expression and secretion of PD1nb in precipitates and culture supernatants of MSCs‐VNP were detected by western blotting in vitro. (C) Schematic diagram of different treatment groups (G1–G4), including saline, MSCs‐VNP (5 × 10^5^ cells per mouse), MSCs‐VNP+PD1nb (5 × 10^5^ cells per mouse and 5 mg/kg PD1nb per mouse), and MSCs‐VNP+MSC‐PD1nb (5 × 10^5^ MSCs‐VNP per mouse and 5 × 10^5^ MSC‐PD1nb per mouse), in a melanoma‐bearing mouse model. (D) Tumor growth profiles after the different treatments (*n* = 5). (E) The overall appearance of tumors in different groups on day 7. Scale bar = 5 mm (*n* = 5). (F) Tumor weight in different groups on day 7 (*n* = 5). (G) PD1nb levels in tumors in different groups on day 7. Scale bar = 10 mm (*n* = 5).

We confirmed that MSCs‐mediated VNP targeted delivery significantly improved tumor targeting of the VNP strain while reducing side effects, thereby enhancing the overall tumor therapeutic effect (Figures 3 and 4). To further examine the synergistic therapeutic effect of the combined treatment of MSCs‐VNP and MSC‐PD1nb, the prepared MSCs‐VNP and MSC‐PD1nb were injected into B16F10 tumor‐bearing mice via the tail vein. The treatment strategy was illustrated in Figure 6C. The results showed that the tumor in the saline group progressed rapidly, whereas the other groups achieved excellent therapeutic outcomes (Figure 6D,E). Encouragingly, the MSCs‐VNP+MSC‐PD1nb group exhibited the most significantly enhanced anticancer effect among all groups. Additionally, on the 7th day posttreatment, the tumor weight of mice administered with MSCs‐VNP+MSC‐PD1nb was only 67.7% of that of mice treated with MSCs‐VNP+PD1nb (Figure 6F). This is understandable, as engineered MSC‐PD1nb were tumor‐targeted and could express and secrete PD1nb if they were alive, resulting in higher intertumoral PD1nb levels compared with simple mixed PD1nb which were shortly degraded (Figure 6G), thus achieving most significant and persistent antitumor effects. Finally, to explore the mechanism underlying the synergistically enhanced therapeutic effect of MSC‐PD1nb, we first assessed tumor apoptosis (Figure S20A,B). The results indicated that synergistic MSC‐PD1nb caused more pronounced tumor cell apoptosis than other regimens. Further H&E staining of tumor sections showed more extensive necrotic areas in tumors from mice treated with MSC‐PD1nb (Figure S20C–E). Correspondingly, we used flow cytometry to examine the expression of Ki67^+^/CD45^−^ (cell proliferation marker) in tumor cells (tumor tissue contains a lot of immune cells, and CD45^−^ cells were considered as tumor cells), and the results revealed that all groups except the saline group effectively inhibited tumor cell proliferation (Figure S21A,B). In addition, the tumor microenvironment is closely related to tumor progression. Thus, we used flow cytometry to assess the tumor infiltration of immune cells after treatment, and found that the number of intertumoral immune cells increased dramatically (Figure S21C,D). These results suggested that higher intertumoral PD1nb levels can be achieved by engineering MSC‐PD1nb to continuously produce and secrete PD1nb within tumors. Thus, combined with MSCs‐VNP, MSC‐PD1nb further synergistically enhances therapeutic efficacy, offering a convenient and long‐lasting treatment strategy.

To determine whether the antitumor efficacy of MSCs‐VNP arose from tumor‐specific mechanisms rather than systemic immune stimulation, and to establish the optimal administration sequence in combination with MSC‐PD1nb, tumor‐bearing mice were treated with nontumor‐targeting bacterial controls (including *E. coli* and inactivated VNP) and sequential administration of MSC‐VNP followed by MSC‐PD1nb 3 days later (Figure S22A). Systemic inflammatory responses, as reflected by serum levels of IL‐6 and IL‐10, were markedly elevated in the nontumor‐targeting bacterial groups compared with the MSC‐VNP group. Despite this heightened systemic immune stimulation, the nontumor‐targeting bacterial groups failed to achieve comparable antitumor efficacy. Conversely, MSC‐VNP treatment yielded significantly superior tumor suppression, as demonstrated by reduced tumor volume and prolonged survival (Figures S22B,C, S23, and S24A). These findings indicated that the therapeutic benefit of MSC‐VNP was attributable to tumor‐specific enrichment rather than nonspecific systemic effects. Notably, sequential administration of MSC‐VNP followed by MSC‐PD1nb exhibited the most potent antitumor activity among all treatment groups, as evidenced by the smallest tumor volume and weight, and the longest median survival (Figure S22B,C; Figure S24A–C). In summary, these findings demonstrated that MSC‐VNP exerted antitumor efficacy through tumor‐specific mechanisms rather than nonspecific systemic immunostimulation, and that sequential administration of MSC‐VNP followed by MSC‐PD1nb represented the optimal regimen for synergistic antitumor activity.

### 2.7 MSCs‐VNP and MSC‐PD1nb Activated the Immune Microenvironment of Tumor

The intertumoral infiltration and activation of immune cells play an important role in antitumor responses to VNP stains. We explored the infiltration of immune cells in the tumor microenvironment following different treatments by FACS, and the experimental procedure is shown in Figure S25. In the tumor microenvironment, macrophages are the predominant immune cells. The results demonstrated that compared with saline treatment, macrophage infiltration rates were significantly increased after MSCs‐VNP‐based treatments (1.46‐ and 1.17‐fold, respectively). Additionally, the percentage of M1‐like macrophages associated with antitumor activity was increased significantly compared with the saline group, while the proportion of M2‐like macrophages with immunosuppressive effects was decreased. Consequently, the M1/M2 ratio increased (1.46‐ and 1.17‐fold, respectively) (Figure 7A–F). Dendritic cells (DCs) are another type of antigen‐presenting cells (APCs) capable of activating antitumor T cells. Therefore, we evaluated DCs infiltration following MSCs‐VNP‐based treatments (Figure 7G,H). As expected, DC levels were significantly increased 6–10‐fold compared with the saline group. These findings suggest that MSCs‐mediated tumor delivery of VNP can establish a foundation for more effective antitumor therapy by recruiting antigen‐presenting and tumor‐killing immune cells (macrophages and DCs).

**FIGURE 7:**
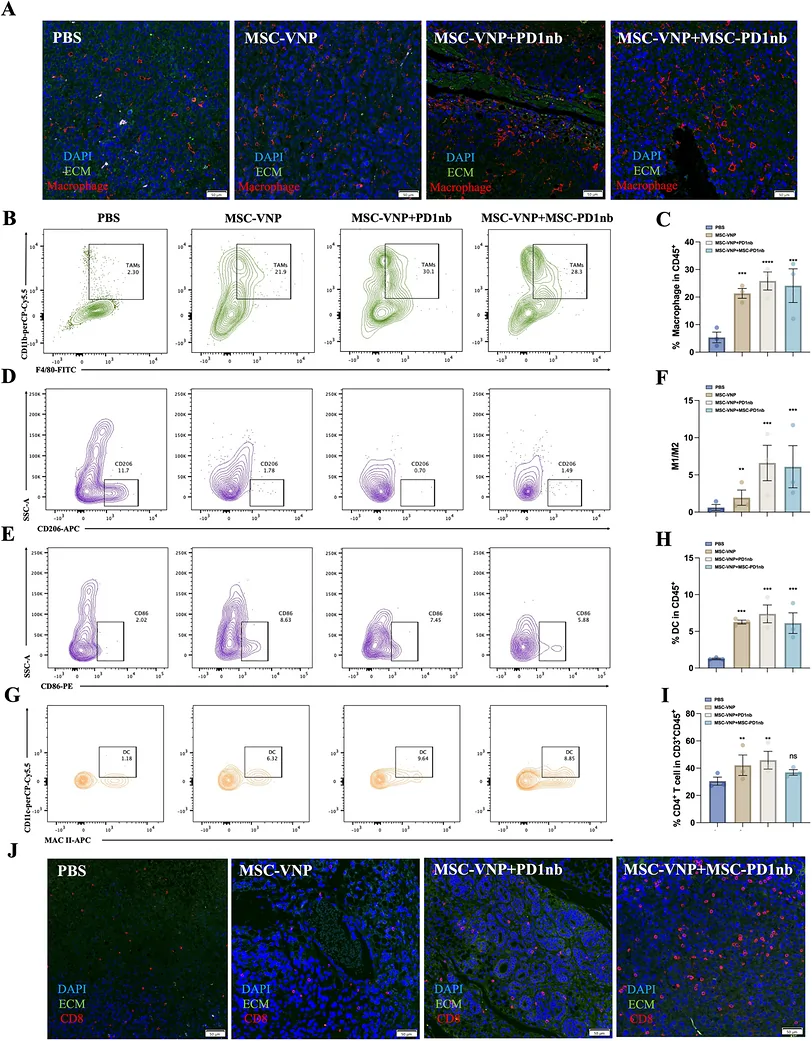
MSCs‐VNP and MSC‐PD1nb activated the immune niche of the tumor. (A) Representative immunofluorescence staining of macrophage infiltration in B16F10 tumor sections (nuclei: DAPI (blue), extracellular matrix: Anti‐ECM (green), and macrophages: F4/80 (red)). Scale bar = 50 µm. (B–F) Representative FACS plots and quantification of total tumor‐infiltrating macrophages, M1‐like macrophages, and M2‐like macrophages (*n* = 3). (G, H) Representative FACS plots and quantification of total tumor‐infiltrating DC cells (*n* = 3). (I) Quantification of tumor‐infiltrating CD4^+^ T cells (*n* = 3). (J) Representative immunofluorescence staining of CD8^+^ T cells in B16F10 tumor sections (nuclei: DAPI [blue], ECM: anti‐ECM [green], CD8^+^ T cells: anti‐CD8 [red]). Scale bar = 50 µm.

Following confirmation of the enhanced therapeutic effect of synergistic MSC‐PD1nb treatment with MSCs‐VNP in vivo, we speculated that MSCs‐mediated intertumoral PD1nb enrichment could reverse the immunosuppressive tumor niche by promoting T lymphocyte activation. CD8^+^ T cells are crucial for exerting anticancer effects, but T cell exhaustion is common in tumors, rendering them unresponsive to ICB therapy [6, 39]. Notably, the proportion of tumor‐infiltrating CD4^+^ T cells showed no significant change among the treatment groups (Figure 7I). In contrast, results showed that synergistic MSC‐PD1nb treatment resulted in significantly higher CD8^+^ T cell infiltration compared with the saline, MSCs‐VNP, or MSCs‐VNP+PD1nb groups (Figure 7J; Figure S26A,B). In contrast, synergistic MSC‐PD1nb treatment reduced the proportion of CD4^+^ T cell infiltration; this is understandable because the proportion of Treg cells (CD4^+^ FOXP3^+^ cells) was significantly reduced (Figure S26C,D). Immunosuppressive CD4^+^ T cells (Tregs) are known to facilitate cancer progression [40]. Our results demonstrated that the proportion of intertumoral Treg cells decreased by 88.3%, 82.5%, and 61.2% compared with saline, MSCs‐VNP, and MSCs‐VNP+PD1nb treatment, respectively (Figure S26C,D). Similarly, this phenomenon was observed in the spleen as well (Figure S27). In contrast, the status of CD8^+^ T cells in the spleen was different from that in the tumor, with no increase in CD8^+^ T cells or IFN^+^CD8^+^ T cells observed after synergistic MSC‐PD1nb treatment (Figure S27A–E). This may be attributed to the fact that PD1nb secreted by MSCs is primarily enriched in the tumor rather than the spleen. In summary, MSCs‐mediated intertumoral proliferation of VNP strains and enrichment of PD1nb not only significantly depleted immunosuppressive M2‐like macrophages and Treg cells in the tumor tissue, but also markedly increased the proportion of antitumor M1‐like macrophages, DC cells, and cytotoxic CD8^+^ T cells. Collectively, these results indicated a reversal of the immunosuppressive tumor microenvironment by MSCs‐VNP in conjunction with MSC‐PD1nb, highlighting their great potential as a potent anticancer therapy.

## 3 Discussion

Oncolytic bacterial therapy has great application potential for treating solid tumors because of its tumor‐targeting capabilities and tumor immune niche activation. However, the severe adverse effects of oncolytic bacteria hamper their clinical applications. We constructed an MSCs‐mediated tumor‐targeted delivery system for the oncolytic bacteria VNP20009 (MSCs‐VNP) and validated the feasibility, safety, and efficacy of this strategy in a melanoma‐bearing mouse model. In addition, engineered MSCs that sustainably secreted PD‐1 nanobodies (MSC‐PD1nb) in combination with MSC‐VNP synergistically markedly improved antimelanoma efficacy by remodeling the immunosuppressive TME. Our study indicates that MSC‐mediated novel combination immunotherapy offers a prospect for expanding safe therapeutic application of oncolytic bacteria in solid cancer.

In clinical trials, oncolytic bacteria are directly administered to humans through intravenous injection [13], leading to the immediate exposure of oncolytic bacteria to the immune system. On the one hand, the oncolytic bacterial strains trigger rapid immune activation, resulting in their swift clearance. On the other hand, bacterial enrichment in tumors mainly relies on proliferation in an anaerobic environment rather than active targeting [12]. Consequently, some strains are randomly distributed to normal organs, causing inflammatory lesions [53]. In a phase I clinical trial of intravenous treatment for sarcoma, VNP was terminated due to its severe adverse effects [13]. Therefore, seeking a safe and effective oncolytic bacteria‐based antitumor therapy is particularly crucial in clinical applications. A variety of MSCs derived from different origins are widely used in regenerative medicine and immune‐imbalanced diseases in basic research and clinical trials due to their distinct advantages. Additionally, MSCs possess strong tumor‐targeting capabilities, and many studies have utilized MSCs to deliver oncolytic adenoviruses (OAds) for the treatment of various tumors [28, 29, 30, 31]. These studies found that MSCs successfully reached the tumor and expressed OAd specifically at the tumor site, with no evidence of OAd infection in other organs (e.g., heart, lung, kidney, spleen). Consistent with these findings, MSCs encapsulated living oncolytic bacteria VNP20009 also successfully homed to the tumor and released VNP strains at the tumor site, whilst constraining VNP strains to the main organs. Direct VNP administration induced severe splenomegaly, liver damage, and elevated inflammatory factor levels. The MSC‐mediated VNP delivery system addressed these challenges. First, MSC‐encapsulated VNP shields the bacteria from immune system exposure, leading to a significant downregulation of CRP and Treg levels in vivo (Figure 4F–H), thereby mitigating the inflammatory responses induced by the strains. Second, the intrinsic tumor‐targeting ability of MSCs facilitates earlier penetration of the strains into tumor tissues (Figure 3E). Finally, the tumor‐homing capability of MSCs substantially minimizes the distribution of VNP to off‐target organs such as the liver, spleen, and lungs (Figure 3G; Figure S11), effectively preventing tissue damage associated with direct bacterial administration. Consequently, the incidence of acute adverse reactions and associated mortality was markedly reduced (Figure 4I). Collectively, the MSC‐mediated VNP delivery system demonstrated enhanced safety and efficiency in tumor‐specific bacterial enrichment, offering a promising avenue for the clinical translation of bacterial therapies in oncology.

Thus, MSCs offer greater accessibility and possess tumor‐targeting capabilities comparable to immune cells, allowing them to serve as an alternative for tumor‐targeted delivery of VNP. Our strategy using MSCs encapsulating living oncolytic bacteria has more advantages than immune cells. Immune cells are theoretically better delivery vehicles, due to their superior tumor‐killing and targeting capabilities. Indeed, a series of studies have demonstrated the effectiveness of using immune cells (macrophages, neutrophils) as bacterial tumor‐targeted delivery vehicles [21, 22, 23, 41, 42]. However, primary immune cells are difficult to extract and expand, which increases costs and limits their clinical application. This challenge is common to all immune cell therapies for tumor treatment [43]. Furthermore, traditional cell‐based cancer therapies or cell carrier therapies require the injection of a large number of cells (10^7^–10^8^ cells in humans) [44, 45, 46]. This means a more cumbersome preparation process and higher economic costs. Therefore, there is an urgent need to reduce the cost of cell therapy and improve its accessibility for clinical tumor treatment. MSCs are easy to culture in vitro and proliferate rapidly, providing a potential solution to the issue of cell sourcing [25, 26, 27]. Notably, MSCs exhibit low MHC expression, and the immune rejection caused by allogeneic MSC infusion is minimal. Stored MSCs from unrelated donors have been widely and safely used in clinical practice [47, 48, 49]. Allogeneic infusion is particularly valuable, as it significantly reduces the cost of cell therapy. Building on these advantages, the utility of MSCs as a targeted delivery platform has been further validated in challenging tumor models, such as nanoengineered MSCs successfully employed for targeted therapy against intramedullary spinal cord tumors [50].

In addition, previous studies have predominantly used MSCs to load traditional anticancer drugs intracellularly [51, 52]. However, achieving therapeutic drug doses requires the injection of large cell quantities (10^7^–10^8^ per mouse). We proposed a therapeutic strategy that employs MSCs to deliver “live” drugs, such as VNP strains. Although each MSC in our study can transport only one VNP strain, a dose of 5 × 10^5^ MSCs‐VNP effectively treated tumors, requiring only 1/100 of the cell quantity needed for traditional cell therapy. Even if MSCs release only a small amount of VNP into the tumor area, they can continue to proliferate and thus achieve therapeutic effects. Moreover, MSCs have excellent proliferation potential and can be infused allogeneically, thus reducing cell therapy costs. The MSCs‐mediated VNP delivery system proposed here is modular, allowing VNP to be replaced with other oncolytic bacteria, such as probiotics. This study demonstrated the effectiveness and universality of MSCs as a universal cell carrier to deliver oncolytic bacteria. Compared with in situ tumors, immune cell infiltration is challenging in subcutaneous tumors like melanoma, and even more so for cell carriers to reach these tumors [52]. Excitingly, our results demonstrated that the MSC‐mediated VNP delivery system exhibits excellent melanoma‐targeting capabilities, suggesting that it will show even stronger targeting in in situ tumors, thereby achieving improved therapeutic outcomes. Additionally, this system applies to metastatic tumors and preoperative adjuvant therapy, offering significant clinical potential.

Thorough tumor treatment requires the combination of multiple treatment methods in clinical practice. After immunotherapy with ICBs, a small proportion of well‐treated patients will experience a series of adverse reactions due to drug accumulation in normal organs [53]. To address this, we constructed genetically engineered MSCs (MSC‐PD1nb) that stably express and secrete PD1nb, concentrating PD1nb production within tumors. This cell line was combined with MSC‐VNP for tumor therapy. Our study indicated that PD1nb secreted by engineered MSCs disrupted PD‐1/PD‐L1 interactions, reversing T cell exhaustion, thereby activating tumor CD8^+^ T cells while suppressing Treg cell differentiation, consistent with previous reports [54, 55]. This novel strategy achieved superior antitumor effects compared with combining PD1nb and MSCs‐VNP for therapy. However, we acknowledge the absence of direct intratumoral T cell exhaustion marker (PD‐1, TIM‐3) and IFN‐γ^+^ CD8^+^ T cell analyses as a limitation. Future studies will incorporate these analyses to further elucidate the underlying mechanisms.

Previous studies have primarily utilized VNP for the expression of PD1nb [21, 22]. However, using bacteria to express monoclonal antibodies presents several challenges. First, the production of therapeutic monoclonal antibodies necessitates posttranslational modifications. Mammalian cell expression systems are capable of performing posttranslational modifications, such as glycosylation, which prokaryotic systems cannot replicate [56]. Second, prokaryotic expression systems carry risks such as endotoxin contamination and inclusion body formation. Studies have demonstrated that recombinant protein drugs expressed in *Escherichia coli* elicit strong immune responses in patients, raising potential safety concerns [57]. Mammalian cell expression systems are capable of producing large, complex proteins with human‐like posttranslational modifications, thereby minimizing immunogenicity [58]. Consequently, compared with prokaryotic expression systems like VNP, monoclonal antibody drugs are better suited for production in mammalian cell expression systems. Additionally, given the clinical advantages of MSC therapy, programming MSCs to express PD1nb may represent a superior alternative. In this study, we successfully engineered MSC‐PD1nb to produce PD1nb, demonstrating enhanced safety compared with previous approaches and verifying its antitumor efficacy in vivo. This approach holds greater potential for clinical application.

However, the MSC‐mediated VNP delivery system has some limitations to be addressed. For example, the uncertainty in the release of intracellularly loaded microorganisms is a common challenge in cell‐based drug delivery systems [59]. To address this, we are developing pH‐responsive bacterial release switches leveraging the acidic TME to spatiotemporally control VNP escape from MSCs. This innovation aims to further enhance tumor‐specific bacterial enrichment while minimizing premature release. Alternatively, a supramolecularly engineered bacterial reactor has been developed to enable sustained lactic acid depletion and oxygen generation, further supporting bacterial activity [60]. These strategies offer valuable insights for optimizing the MSC‐based delivery system.

In summary, our results provided the first evidence that MSCs can function as a “Trojan horse” and antitumor antibody producer, aiding VNP strain accumulation and constantly releasing antitumor antibody within tumors to collaboratively achieve better anticancer efficacy. This system improves the biosafety and biocompatibility of oncolytic bacteria‐based cancer therapy by limiting bacterial xenobiotic stimulation. The MSCs‐mediated VNP delivery and antitumor antibody production system may open up new strategies for cell‐ and microbiome‐mediated antitumor therapies.

## 4 Experimental Section

### 4.1 Cell Culture

Human umbilical cord‐derived MSC cell line, MSCs‐EGFP cell line (MSCs stably expressing green fluorescent protein), RAW 264.7 mouse macrophage cells, B16F10 mouse melanoma cells, 293T human embryonic kidney cell line, HGC‐27 (human gastric cancer cells) and A549 (human lung cancer cells) were obtained from laboratory archives. All cell lines were cultured in Dulbecco's modified Eagle's medium (DMEM) with 10% FBS and 100 µg/mL penicillin/streptomycin under 5% CO_2_ at 37°C. RAW264.7 cells were stimulated with 600 ng/mL LPS for 24 h to obtain M1‐type RAW264.7 cells. All procedures involving human subjects were approved by the Research Ethics Committee Board of Nanjing Drum Tower Hospital.

### 4.2 Bacterial Strains and Plasmid Transformation

Attenuated *Salmonella typhimurium* VNP20009 (abbreviated VNP) and *E. coli* were preserved in our laboratory. Engineered strains VNP‐DsRed (transformed with a plasmid expressing DsRed) were obtained by plasmid electrotransformation. Specifically, the constructed plasmids were electroporated into the VNP strain (1.6 kV; 200 Ω; 25 µF), with the empty vector plasmid used as a control strain. All strains were cultured using LB solid or liquid medium.

### 4.3 Preparation of VNP‐Loaded MSCs (MSCs‐VNP)

The VNP strains were resuspended in PBS, and the OD600 was adjusted to 1.0. Subsequently, it was determined that the VNP with OD600 = 1.0 corresponded to a VNP concentration of 3 × 10^8^ CFU mL^−1^. MSCs were cultured in serum‐free medium for 24 h, then incubated with VNP at an MOI of 100 at 37°C in 5% CO_2_ for various durations. Subsequently, the MSCs were washed 2–3 times with PBS to remove most extracellular VNP. Subsequently, 50 µg/mL gentamicin (Sigma‐Aldrich, 1405‐41‐0, St. Louis, USA) was added for 1 h to eliminate free extracellular strains without significantly affecting intracellular strains. Finally, MSCs loaded with VNP (MSCs‐VNP) were obtained after 2–3 washes with PBS. For in vitro experiments, freshly prepared MSCs‐VNP were used within 2 h; for in vivo experiments, within 3 h.

The cells were centrifuged at 9000 rpm for 5 min, and the bacterial pellet was resuspended in 2.5% glutaraldehyde. After stationary fixation for at least 30 min at room temperature in the dark, the electron microscopy section samples were prepared and photographed.

### 4.4 Fluorescence Microscopy Imaging

VNP and inactivated VNP (incubated with 4% paraformaldehyde for 1 h) were initially stained with membrane dye FM1‐43 (Invitrogen, F35355, Carlsbad, USA) for 5 min. MSCs and RAW264.7 were then co‐cultured with VNP and inactivated VNP for different times. Extracellular were washed and 50 ug/mL gentamicin was added for 1 h. After removing extracellular VNP, cells were washed three times with PBS, fixed with 4% paraformaldehyde for 30 min, and permeabilized with 0.5% Triton X‐100 (Sigma‐Aldrich, 648462). After washing with PBST, Alexa Fluor 488 phalloidin (Beyotime, C2201S, Shanghai, China) was added and incubated for 1 h, followed by DAPI (Beyotime, C1005) for nuclear staining. Images were acquired using a microscope (Carl Zeiss, Axioplan 2, Oberkochen, Germany).

### 4.5 Electron Microscopy Imaging

For transmission electron microscopy (TEM), the prepared MSCs and MSCs‐VNP (MOI = 100:1 for 1 h) were collected by centrifugation. Subsequently, the cells were prefixed in 2.5% isopropanol at 4°C for 2–4 h. The samples were suspended in 1% agarose, fixed with 1% osmium tetroxide in 0.1 M phosphate buffer (pH 7.4) at room temperature for 2 h, and washed with 0.1 M phosphate buffer (pH 7.4). After fixation, samples were dehydrated with a graded ethanol series, embedded in Epon812, and polymerized at 60°C for 48 h. Ultrathin sections (60–80 nm) were cut using a microtome and double‐stained with 2% uranyl acetate and 2.6% lead citrate for TEM observation. Intracellular bacterial morphology was imaged using a Hitachi HT‐7700 TEM.

### 4.6 MSCs Viability Assays

The viability of VNP‐loaded MSCs was assessed using trypan blue staining (Invitrogen, T10282). Briefly, the cell suspension was mixed with 0.4% trypan blue solution, and 20 µL of the suspension was used to count viable and nonviable cells.

### 4.7 Determination of the Number of VPN in MSCs

To determine the number of VNP strains loaded in MSCs, MSCs‐VNP were lysed with 0.5% Triton X‐100 (Sigma‐Aldrich, 648462) for 15 min to release intracellular VNP. The lysate was diluted, plated on LB agar supplemented with kanamycin, and incubated overnight. Viable VNP colonies taken up by MSCs were then counted.

### 4.8 Quantitative Real‐Time PCR Assay

Total RNA was extracted using TRIzol reagent (Vazyme, China), and cDNA was synthesized using a reverse transcription kit (Vazyme). Relative mRNA levels were amplified using AceQ qPCR SYBR Green Master Mix (Vazyme, Q221‐01) according to the manufacturer's instructions. Primers were synthesized by GenScript (Nanjing, China). Quantitative real‐time PCR was conducted on a QuantStudio 3 real‐time fluorescence PCR instrument.

### 4.9 MSCs Chemotaxis Assay

MSCs chemotaxis assays were conducted using Transwell inserts with 8 µm pores (Corning, 07‐200‐82, NY, USA). The lower chambers were seeded with 3 × 10^4^ B16F10, HGC‐27, or A549 cells per well in DMEM (controls contained only DMEM without cells), supplemented with 50 µg/mL gentamicin and 10% fetal bovine serum. The upper chambers were seeded with 5 × 10^5^ MSCs or MSCs‐VNP per well and incubated at 37°C for 12 h. After incubation, the upper chambers were removed, nonmigrated cells on the upper membrane surface were removed with a cotton swab, and migrated cells on the lower membrane surface were fixed in 4% paraformaldehyde for 30 min, followed by staining with crystal violet (Sigma‐Aldrich, C0775) for 45 min at room temperature. After washing with PBS, the upper chambers were photographed under an orthogonal fluorescence microscope (Zeiss).

### 4.10 Bacterial Release Assays

To assess VNP release from MSCs, a VNP‐DsRed strain expressing red fluorescent protein (DsRed) was constructed as previously described. The VNP‐DsRed and MSCs were cocultured at a MOI of 100:1 for 1 h. Subsequently, 50 µg/mL gentamicin (Sigma‐Aldrich, 1405‐41‐0) was added for 1 h to eliminate free extracellular strains without significantly affecting intracellular strains. Finally, MSCs‐VNP‐DsRed were washed 5–6 times with PBS and cultured in DMEM supplemented with 10 µg/mL gentamicin (sufficient to inhibit VNP proliferation but not to kill them) and 10% serum. Meanwhile, a live cell imaging system (LUX 3, Axion, Shanghai, China) was used to track VNP‐DsRed in MSCs for 24 h.

To accurately quantify VNP release, MSCs‐VNP cells were cultured in 3.0 µm Transwell plates (pores allowed VNP passage but not MSCs). MSCs‐VNP were inoculated in the upper chamber, and 10 µg/mL gentamicin was added to the lower chamber. The transwell plate was placed at 37°C for 18 h. The supernatant from the lower chamber was collected, plated on LB agar, and incubated to count viable VNP released by MSCs.

### 4.11 Tumor Cell Apoptosis Detection

B16F10 cells were co‐cultured with either unprocessed VNP or released VNP strains for 16 h at an MOI of 200. All B16F10 cells were removed, washed, and resuspended in 100 µL binding buffer, then stained with 1 µL Annexin V‐APC (1 mg/mL) and 1 µL PI‐PE (1 mg/mL) in the dark for 30 min at 4°C. The stained B16F10 cells were analyzed using FACS (NovoCyte Flow Cytometer (ACEA@)).

### 4.12 In Vivo Biodistribution

MSCs‐EGFP and VNP‐DsRed were prepared. MSCs‐EGFP, VNP‐DsRed, and MSCs‐VNP (expressing EGFP and DsRed) were suspended in 150 µL saline and injected via the tail vein into melanoma model mice. After euthanasia at the predetermined time points, tumors and other organs (heart, lungs, spleen, liver, and kidneys) were dissected and lysed with 1% Triton X‐100 at 4°C for 1 h. To determine VNP distribution, the tissue lysate was diluted, plated on LB agar, and incubated overnight for colony counting. A separate portion of the tissue lysate was used to detect VNP‐DsRed distribution by FACS. Concurrently, tissue sections were prepared for fluorescence microscopy to detect VNP‐DsRed distribution.

MSCs‐EGFP, VNP‐DsRed, and MSCs‐VNP (expressing EGFP and DsRed) were suspended in 150 µL of saline and injected into melanoma model mice via the tail vein. The fluorescence signals of MSCs‐EGFP and VNP‐DsRed in the tumors of mice after different treatments were identified using an in vivo situ cell dynamic imaging system (IVIM, Waltham, MA, USA).

### 4.13 Animal Model

To establish a melanoma model, C57BL/6J mice (6–8 weeks, female) were injected with B16F10 cells (5 × 10^5^ cells in 100 µL of PBS per mouse) into the right flank. PD1nb was purified from MSC‐PD1nb in our laboratory. When the tumors grew to 50–160 mm^3^(≈10–12 days). PD1nb (5 mg/kg), VNP (5.0 × 10^5^ CFU per mouse), and cells (5.0 × 10^5^ cells per mouse), including MSCs, MSCs‐VNP, and MSC‐PD1nb, were injected via the tail vein into melanoma‐bearing mice. The mice's body weight and tumor volume were measured 3–4 times a week. Tumor volumes (V, mm^3^) were determined using the formula *V* = *a* ^2^ *b* × 0.52. Here, “*a*” represents the shorter diameter and “*b*” represents the longer diameter. Subsequently, after a specific time of administration, the mice were euthanized, and tumors, heart, lungs, spleen, liver, kidneys, and peripheral blood were obtained. The whole peripheral blood samples were used for subsequent ELISA or safety analysis. Subsequently, tumors and other organs were sent to Wuhan Servicebio Technology Co., Ltd. (Wuhan, China) for embedding, sectioning, and H&E staining. To plot the survival curve, other tumor‐bearing mice were monitored daily. Death was defined as comprising natural death and humane‐endpoint euthanasia. Natural death was recorded as spontaneous death without intervention. When signs of adverse effects (pain, apathy, necrotic tumor) were observed or the humane endpoint (tumor weight equal to 10% of mouse body weight, or tumor volume reaching 1500 mm^3^) was reached. All animal experiments complied with the ethical guidelines of the Animal Care and Use Committee of Nanjing Drum Tower Hospital.

For the sequential treatment experiments, melanoma‐bearing mice were randomly assigned to seven groups: saline, E. coli (5.7 × 10^5^ CFU per mice), inactivated VNP (5.7 × 10^5^ CFU per mice), VNP (5.7 × 10^5^ CFU per mice), MSCs‐VNP (5 × 10^5^ cells per mice), MSCs‐VNP + MSC‐PD1nb (5 × 10^5^ cells per mice), and MSCs‐VNP followed by MSC‐PD1nb (5 × 10^5^ cells per mice) 3 days later. All treatments were administered via the tail vein. *E. coli* was preserved in our laboratory. Inactivated VNP was prepared by incubation with 4% paraformaldehyde for 1 h. Serum IL‐6 and IL‐10 levels were measured by ELISA on day 3 after treatment (*n* = 6). Tumor volume, tumor weight, and survival were assessed as described above, with survival monitored until day 42.

### 4.14 Engineered MSC‐PD1nb Construction and Screening

The PD1nb coding sequence was synthesized in vitro by GenScript and inserted into the pcDNA3.1 plasmid. All plasmids were confirmed by sequencing before subsequent experiments. To generate MSC‐PD1nb, 1 × 10^6^ s‐generation (P2) MSCs were placed in 100 µL of nucleofection solution (cat. no. PT‐2501, Lonza, Germany), electroporated with 2 µg of plasmid in a cuvette using the Nucleofector apparatus (Lonza) with the U‐23 program (for high‐transfection efficiency). Electroporated MSCs were immediately removed from the cuvette, seeded in a 10 cm culture dish, and incubated at 37°C with 5% CO_2_. Stable clones were screened using 1.2 µg/mL G418 disulfate (Solarbio, IG0010, Beijing, China). Finally, engineered MSCs (MSC‐PD1nb) were obtained by G418 selection after 2 weeks, and each MSC‐PD1nb clone represents a cell line. Once each MSC‐PD1nb clone reached 90% confluence, the supernatant was collected, and PD1nb levels were quantified by ELISA.

### 4.15 Enzyme‐Linked Immunosorbent Assay (ELISA)

Human and mouse PD‐1 proteins were coated on 96‐well plates (Corning, Cat. No.: 9018) at 50 ng/well and incubated at 4°C overnight. The antigen‐coated plates were blocked with 200 µL blocking buffer for 1 h at 37°C, followed by washing with PBST. MSC‐PD1nb culture supernatants and tumor lysates were added and incubated at 37°C for 1.5 h. Finally, protein levels were detected according to the method described previously [27].

### 4.16 Flow Cytometry

Tissues from tumor‐bearing mice were collected on day 7 after treatment. The spleen was homogenized with 1 mL PBS to obtain a single‐cell suspension. Tumor tissues were minced and digested with digestion buffer (1 mg/mL collagenase I, 200 µg/mL DNase I, 1 mg/mL collagenase IV) at 37°C for 40 min. Cells were then lysed with erythrocyte lysis buffer on ice for 1 min. To obtain single‐cell suspensions, the cell mixture was passed through a 40 µm cell strainer. Single‐cell suspensions were stained with viability dye (BD, 564407) followed by staining with the following antibodies: CD45‐BV510 (BD, 740 131), CD11b‐FITC (BD, 561 684), CD11c‐PE‐Cy7 (BD, 558 079), MHCII‐PerCP‐Cy5.5 (BD, 562 363), F4/80‐BV421 (BD, 566 787), CD8‐PE‐Cy7 (BD, 552 877), CD4‐PerCP‐Cy5.5 (BD, 550 954), and CD206‐APC (Invitrogen, 17‐2061‐82) at 4°C for 30 min. After surface staining, cells were fixed and permeabilized using a fixation/permeabilization kit according to the manufacturer's instructions, and then stained with intracellular antibodies including Ki67‐BV421 (BD, 562 899), IFN‐γ‐APC (BD, 562 018), and Foxp3‐PE (BD, 563 101) at 4°C for 30 min in the dark. Stained cells were analyzed by flow cytometry (BD Canto II).

### 4.17 Statistical Analysis

The data were expressed as the mean ± SD as specified. Mean differences were compared using *t*‐tests (for two groups) or one‐way analysis of variance (for multiple groups). A value of *p <* 0.05 was regarded as statistically significant. Data were analyzed using GraphPad Prism 8.3 software. (****p < 0.0001, ***p <0.001, **p<0.01, *p<0.05.)

## 5 Supporting Information

Supporting Information is available from the Wiley Online Library or from the author.

## Author Contributions

Yong Liu and Bin Wang: conceptualization and funding, methodology. Yanning Wang and Tianyun Gao: data curation, writing – original draft preparation, software. Feifei Huang: data curation, software. Yaping Wang, Mingmei Yang, Congwang Xu: software, validation. Dong Chen, Shuo Liu, Yuanyuan Xie: writing – reviewing and editing. All authors have read and approved the final manuscript.

## Funding Information

This work was supported by the National Key Research and Development Program of China, No. 2017YFA0104304; the National Natural Science Foundation of China, No. 81571213, 82070459, 82270701 (to Bin Wang), 81800583 (to Yuanyuan Xie), Key Project of Jiangsu Province (Grant No.BE2020765) (Bin Wang), Nanjing Medical Science and Technique Development Foundation (ZKX20016) (Bin Wang); Jiangsu Provincial Plan for Mass Entrepreneurship and Innovation (2019) (Bin Wang), and Project of Modern Hospital Management and Development Institute, Nanjing University/Aid project of Nanjing Drum Tower Hospital Health, Education & Research Foundation (NDYG2020030) (Bin Wang). Nanjing Municipal Health Science and Technology Development Special Fund Project [No. YKK23108 (Yuanyuan Xie)]; Nanjing Municipal Health Science and Technology Development Special Fund Project(No. YKK23089) (Shuo Liu).

## Ethics Statement and Consent to Participate

The study involving human umbilical cord‐derived MSCs was conducted according to the guidelines of the Declaration of Helsinki and approved by the Research Ethics Committee Board of Nanjing Drum Tower Hospital (approval no. 2017–161‐01, Project: “Utilization of Clinical Patient Samples for Stem Cell Extraction and Research in Regenerative Medicine”, Date: 2017‐12‐26). Written informed consent was received from all donors of human umbilical cord.

All animal experiments were performed in the light of the Guide for the Care and Use of Laboratory Animals from the National Institutes of Health, and this study was approved by the Institutional Animal Care and Use Committee of Experimental Animal Center of Nanjing Drum Tower Hospital (approval no. 2022AE01040, Date: 2022‐6‐25).

## Consent for Publication

The authors have nothing to report.

## Conflicts of Interest

The authors declare no conflicts of interest.

## Supporting information




**Supporting file 1**: mco271142‐sup‐0001‐Video S1.avi

**Supporting file 2**: mco271142‐sup‐0002‐Video S2.mp4

**Supporting file 3**: mco271142‐sup‐0003‐Video S3.mp4

**Supporting file 4**: mco271142‐sup‐0004‐Video S4.mp4

**Supporting file 5**: mco271142‐sup‐0005‐SuppMat.docx

## Data Availability

All data generated or analyzed during this study are included in this published article. The datasets used and/or analyzed during the current study are available from the corresponding author upon reasonable request.
