# Supplementary material for: Mesenchymal Stromal Cells‐Mediated Tumor‐Targeted Delivery of Oncolytic Bacteria Combined With Immune Checkpoint Blockade Against Melanoma
Source: MedComm (2020). 2026 Nov 13;7(11):e71142. doi: 10.1002/mco2.71142 (PMC13761283; doi:10.1002/mco2.71142)
Supplement: Supplementary file 1 [file MCO2-7-e71142-s005.docx]

**Mesenchymal Stromal Cells-mediated Tumor-Targeted Delivery of Oncolytic Bacteria Combined with Immune Checkpoint Blockade against Melanoma**

*Yanning Wang^1#^, Tianyun Gao^1#^, Feifei Huang^1#^, Yaping Wang^2^, Mingmei Yang^2^, Congwang Xu^2^, Dong Chen^1^, Shuo Liu^1^, Yuanyuan Xie^1^, Yong Liu^3^*, Bin Wang^1,2^**

1 Clinical Stem Cell Center, Nanjing Drum Tower Hospital, The Affiliated Hospital of Nanjing University Medical School, Nanjing, 210000, China.

2 Clinical Stem Cell Center, Nanjing Drum Tower Hospital, Clinical Medical College of Traditional Chinese and Western Medicine, Nanjing University of Chinese Medicine, Nanjing, Jiangsu Province, 210009, China.

3 Department of Laboratory Medicine, Nanjing Drum Tower Hospital, Clinical Medical College of Traditional Chinese and Western Medicine, Nanjing University of Chinese Medicine, Nanjing, Jiangsu Province, 210008, China.

^#^ Y. Wang, T Gao and F Huang contributed equally to this work.

* Correspondence should be addressed to Yong Liu (liuyongbio@126.com) and Bin Wang ([wangbin022800@126.com](mailto:wangbin022800@126.com)).

*Bin Wang, PhD

Clinical Stem Cell Center, the Affiliated Drum Tower Hospital of Nanjing University Medical School, 321 Zhongshan Road, Nanjing 210008, China.

Telephone: 86-025-68182508

Fax: 86-025-68183333

E-mail: [wangbin022800@126.com](mailto:wangbin022800@126.com)

* Yong Liu, PhD

Department of Laboratory Medicine, Nanjing Drum Tower Hospital, Clinical Medical College of Traditional Chinese and Western Medicine, Nanjing University of Chinese Medicine, Nanjing, Jiangsu Province, 210008, China.

Telephone: 86-025-68182508

Fax: 86-025-68183333

E-mail: liuyongbio@126.com

**Supporting Information**

**Figure S1. Exploration of constructing VPN-loaded MSCs.**

A. Gentamicin (50 μg/mL) completely eliminated the VNP within 60 min.

B, C. Representative plates images and quantification of intracellular VNP number at different MOI. (n = 3).

D, E. Representative plates images and quantification of intracellular VNP number at different coculture condition and time. (n = 3).

F. The gene expression levels of strains encapsulated in MSCs were measured by qPCR (n=6).

**Figure S2. Mechanism of MSCs encapsulating VNP.**

A. The fluorescent images of VNP (FM1-43, green). Scale bar = 100 μm (left) or 50 μm (right).

B, C. The fluorescent images of MSC-VNP and RAW-VNP; VNP (green), actin (red), DAPI (blue). Scale bar = 100 μm (left) or 50 μm (right).

**Figure S3. Status of MSCs-VNP.**

A. The survival of MSCs cocultured with VNP at different time points (n=3).

B. The viability of MSCs after loading VNP; the media supplemented with gentamicin (n=3).

**Figure S4. Apoptosis of B16F10.**

The apoptosis of B16F10 after incubation with VNP and MSCs-released VNP strain (R-VNP) after cryopreservation in liquid nitrogen for 15/30 /60 days (n=3).

**Figure S5. Representative images of chambers.**

**Figure S6. Construction and characterization of VNP-Dsred.**

Plasmid map of DsRed expression and representative fluorescent images of VNP-DsRed. Scale bar = 20 μm (left) or 2 μm (right).

**Figure S7. Dynamic video of VNP release from MSCs. Scale bar: 5 μm.**

**Figure S8. The growth profiles and antitumor efficacy of MSCs-released VNP under simulated tumor microenvironment.**

A. Growth curves of VNP and MSCs-released VNP strain (R-VNP) in medium with pH = 6.7 or hypoxia (n = 3).

B. The apoptosis of B16F10 after incubation with VNP and R-VNP in medium with pH = 6.7 or hypoxia (n = 3).

**Figure S9. Infiltration sites and retention duration of VNP.**

A. Representative fluorescent images of VNP in tumors at different time points (nuclei: DAPI (blue), VNP: FM1-43(green)). Scale bar = 100 μm.

B. C. Representative fluorescent images of VNP infiltration at 48h and long-term retention within distinct tumor compartments (nuclei: DAPI (blue), VNP: FM1-43(green)). Scale bar = 100 μm.

**Figure S10. VNP loading into MSCs reduced the off-target effects of VNP.**

Representative fluorescent-field pictures of the lung burden of VNP at 48h after administrations in the melanoma model. Scale bars: 100 μm or 25 μm.

 **Figure S11. MSC-VNP reduce bacterial off-targeting in normal organs.**

A, B. Representative FACS plots and quantified data of organ burden of VNP at 48h after administrations in the melanoma model. (n = 4).

C, D. Representative images of VNP culture plates at 48h and quantified data of VNP colonies on LB agar plates from homogenized organ tissues. (n = 4).

**Figure S12. MSCs-VNP show no detectable acute toxicities.**

A. Body weight 0, 1 and 3 days after different treatments in the healthy mouse. (n = 8 or 10).

B. H&E-stained images of organs from healthy mice 3 days post-treatment. Scale bars: 100 μm.

**Figure S13. MSCs-VNP show no detectable chronic toxicities.**

A, B. Representative images of spleen and ration of spleen weight/body weight at day 14/28 after different treatments. Scale bar = 1 cm. (n = 5).

C. Body weight changes after different treatments in the healthy mouse. (n = 5).

D, E. Serum alanine transaminase (ALT), aspartate transaminase (AST), C-reactive protein (CRP), IL-6, and IL-10 levels of mice at day 14/28 after different treatments. (n = 3).

**Figure S14.** Representative FACS plots and the percentage of Tregs in the peripheral blood at day 14/28 after different treatments. (n = 3).

**Figure S15.** H&E-stained images of organs from healthy mice 14/28 days post-treatment. Scale bars: 100 μm.

**Figure S16. The long-term VNP biodistribution in normal organs.**

A, B. Representative FACS plots and quantified data of 28 days after administrations, the organ burden of VNP-Dsred in the healthy mouse. (n=3).

C. The organ burden of VNP at Day14/28 after administrations in healthy model (nuclei: DAPI staining (blue), VNP (green)). Scale bar = 25 μm.

**Figure S17.** **Antitumor effect and body weight after MSCs-VNP treatment.**

A. The overall appearance picture of tumor in different groups at day 7. Scale bar = 5 mm. (n = 7 or 8).

B. Body weight 0, 1, 3 and 7 days after different treatments in melanoma bearing mouse model. (n = 8 or 10).

**Figure S18.**  Representative fluorescence images of apoptosis staining in B16F10 tumor (green) after different treatments at day 7 (nuclei: DAPI (blue), VNP-Dsred (red)). Scale bar = 100 μm (upper) or 25 μm (lower).

**Figure S19. Characterization of MSC-PD1nb**

A. Affinity of PD1nb secreted by MSCs or 293T cells for PD1 protein (human and mouse), determined by noncompetitive ELISA.

B. Quantification of released PD1nb levels from all MSC clones. (n = 3).

C. PD1nb levels released by screened MSCs at different passages. (n = 3).

**Figure S20. MSC-PD1nb significantly inhibited tumor growth.**

A, B. Representative FACS plots and quantification of the tumor apoptosis levels at day 7 after different treatments. (n = 3).

C, D. Representative H&E images and quantification of the tumor necrosis area in different treatment groups at day 7. Scale bars: 1 mm or 200 μm. (n = 3).

E. Body weight 0, 1, 3 and 7 days after different treatments in melanoma bearing mouse model. (n = 5).

**Figure S21. MSC-VNP suppressed tumor proliferation and enhanced immune cells infiltration.**

A, B. The proliferation of tumor cells (CD45-) was detected by Ki67 staining and flow cytometry. (n = 3).

C, D. Representative FACS plots and quantification of tumor-infiltrating immune cells (CD45^+^) at day 7 after different treatments. (n = 3).

**Figure S22. Antitumor efficacy of sequential MSC‑PD1nb administration.**

A. Schematic diagram of melanoma mouse model in different treatments including saline, E. coli (5.7 × 10^5^ CFU per mouse), inactivated VNP (5.7 × 10^5^ CFU per mouse), VNP strains (5.7 × 10^5^ CFU per mouse), MSCs-VNP (5 × 10^5^ cells per mouse), MSCs-VNP+MSC-PD1nb (5 × 10^5^ MSCs-VNP per mouse and 5 × 10^5^ MSC-PD1nb per mouse) and MSCs-VNP+ Continuing MSC-PD1nb (5 × 10^5^ MSCs-VNP per mouse and 5 × 10^5^ MSC-PD1nb per mouse 3 days later).

B. Tumor growth profiles after the different treatments. (n = 8).

C. Survival curves of melanoma-bearing mice. (n = 8).

**Figure S23. MSCs-VNP attenuated systemic immune responses.**

IL-6, and IL-10 levels of mice at day 3 after different treatments. (n = 6).

**Figure S24. Sequential MSC-PD1nb treatment enhanced antitumor efficacy.**

A. The overall appearance picture of tumor in different groups at day 7. Scale bar = 5 mm. (n = 8).

B. Tumor weight in different groups at day 7. (n = 8).

C. Representative H&E images in different treatment groups at day 7. Scale bars: 200 μm. (n = 3).

**Figure S25. Representative gating strategy.**

**Figure S26. The change of T cells in Tumor**

A, B. Representative FACS plots and quantifications of tumor-infiltrating CD8^+^ T cells, CD4^+^ T cells. (n = 3).

C, D. Representative FACS plots and quantifications of tumor-infiltrating Tregs. (n = 3).

**Figure S27. The change of T cells in spleen**

A-G. Representative FACS plots and quantification of CD8^+^T cell, CD4^+^T cell, activated CD8^+^T cell, Tregs. (n = 3).

**Supplementary Table. Quality Control Criteria for MSCs-VNP Preparation and Characterization.**

| **Category** | **Quality Attribute** | **Methods** | **Criteria** |
| --- | --- | --- | --- |
| MSCs Identity | Phenotype | Flow cytometry | ≥95% positive for CD105, CD73, CD90; ≤2% positive for CD34, CD45, HLA-DR |
|  | Viability (pre-loading) | Trypan blue staining | ≥95% viable cells |
| VNP Identity | Viability | OD600 and colony counting | OD600=1.0 (3 × 10⁸ CFU/mL) |
|  | Purity | Streak on LB agar | No contamination with other bacteria |
| Preparation Process | Co-culture MOI | Ratio of VNP to MSCs | MOI = 100 |
|  | Co-culture duration | Incubation time in serum-free medium at 37°C | 1 hour |
|  | Extracellular bacteria removal | Gentamicin (50 μg/mL for 60 min) | No colony growth on LB agar |
|  | Loading efficiency | Fluorescence microscopy, flow cytometry, qPCR, colony counting | >100CFU/100 cells |
|  | MSC viability (post-loading) | Trypan blue staining | >90% viability after loading; >50% viability at 24 h |
| Functional Attributes | Tumor chemotaxis | Transwell assay | No significant reduction in migrated cell number compared to unloaded MSCs |
|  | VNP release kinetics | Live-cell imaging, colony counting | First release observed at 9 h; complete release by 18 h; |
|  | Tumor cell killing activity | Annexin V/PI staining by flow cytometry | ≥30% apoptotic cells at 18h |
| Stability and Consistency | Consistency | Replicate preparations tested | Variation ≤10% for key parameters (loading, viability, release timing) |
|  | Cryopreservation stability | Freeze-thaw cycle; assess viability and release | Viability ≥90% of pre-freeze; release profile similar to fresh MSC-VNP |
